# Risk assessment of latent tuberculosis infection through a multiplexed cytokine biosensor assay and machine learning feature selection

**DOI:** 10.1038/s41598-021-99754-3

**Published:** 2021-10-15

**Authors:** Heather M. Robison, Cole A. Chapman, Haowen Zhou, Courtney L. Erskine, Elitza Theel, Tobias Peikert, Cecilia S. Lindestam Arlehamn, Alessandro Sette, Colleen Bushell, Michael Welge, Ruoqing Zhu, Ryan C. Bailey, Patricio Escalante

**Affiliations:** 1 Department of Chemistry, University of Michigan, 930 North University Avenue, Ann Arbor, MI USA; 2 Department of Statistics, University of Illinois Urbana-Champaign, 725 South Wright Street, Champaign, IL USA; 3 Department of Immunology, Mayo Clinic, 200 First Street SW, Rochester, MN USA; 4 Department of Laboratory Medicine, Mayo Clinic, 200 First Street SW, Rochester, MN USA; 5 Division of Pulmonary and Critical Care Medicine, Department of Medicine, Mayo Clinic, 200 First Street SW, Rochester, MN 55905 USA; 6 Division of Vaccine Discovery, La Jolla Institute for Immunology, La Jolla, CA USA; 7 Department of Medicine, University of California San Diego, La Jolla, CA USA; 8 National Center for Supercomputing Applications, University of Illinois at Urbana-Champaign, 1205 W. Clark St., Urbana, IL USA

**Keywords:** Biomarkers, Diagnostic markers, Predictive markers, Computational biology and bioinformatics, Machine learning

## Abstract

Accurate detection and risk stratification of latent tuberculosis infection (LTBI) remains a major clinical and public health problem. We hypothesize that multiparameter strategies that probe immune responses to *Mycobacterium tuberculosis* can provide new diagnostic insights into not only the status of LTBI infection, but also the risk of reactivation. After the initial proof-of-concept study, we developed a 13-plex immunoassay panel to profile cytokine release from peripheral blood mononuclear cells stimulated separately with Mtb-relevant and non-specific antigens to identify putative biomarker signatures. We sequentially enrolled 65 subjects with various risk of TB exposure, including 32 subjects with diagnosis of LTBI. Random Forest feature selection and statistical data reduction methods were applied to determine cytokine levels across different normalized stimulation conditions. Receiver Operator Characteristic (ROC) analysis for full and reduced feature sets revealed differences in biomarkers signatures for LTBI status and reactivation risk designations. The reduced set for increased risk included IP-10, IL-2, IFN-γ, TNF-α, IL-15, IL-17, CCL3, and CCL8 under varying normalized stimulation conditions. ROC curves determined predictive accuracies of > 80% for both LTBI diagnosis and increased risk designations. Our study findings suggest that a multiparameter diagnostic approach to detect normalized cytokine biomarker signatures might improve risk stratification in LTBI.

## Introduction

Tuberculosis (TB) is a pervasive and devastating infectious disease that led to 1.2 million deaths in 2019^1^. Beyond the acute morbidity of the disease, it also can manifest as an asymptomatic infection known as latent tuberculosis (LTBI), which greatly complicates disease management. Approximately 1.7 billion people are estimated to have this quiescent infection state^1–3^. Among this population, 5–10% are estimated to develop an active, transmissible, and potentially lethal TB infection^4–6^. LTBI is treatable with prolonged antibiotic regimens, but potential drug-related toxicities and treatment non-adherence issues obviate the need to identify patients most likely to benefit from these therapies. To properly diagnose individuals with LTBI, and more specifically those with high reactivation potential, improved diagnostic tools are needed. While current assays for TB, including the Tuberculin Skin Test (TST) and Interferon-Gamma Release Assays (IGRAs), reveal previous exposure to TB, they offer limited benefit for LTBI diagnosis and reactivation risk stratification^7^. There is a growing consensus that this latent infection and the conditions that foster reactivation include complex changes in the immunological landscape that may be independent of the pathogen itself^8–14^. Therefore, multiparameter strategies that probe immune (dys)regulation in response to TB-specific and non-specific antigen challenge may provide new diagnostic insights into not only the status of LTBI infection, but also the risk of reactivation.

Previously, our groups described an approach to multiplexed cytokine profiling of supernatants from stimulated peripheral blood mononuclear cells (PBMCs) using arrays of silicon photonic microring resonators^15^. This workflow involved five different TB-specific and non-specific stimulation conditions, and the quantification of seven cytokines. Using a precision normalization approach to correct for individual patient heterogeneity and a bioinformatic feature selection approach, we identified multi-biomarker signatures that preliminarily correlated with LTBI status and elevated reactivation potential. This study suggested relevant biomarkers beyond interferon-gamma (IFN-γ), which is the cytokine detected in IGRAs, and also highlighted the importance of precision normalization using non-TB-related antigen challenges as a way of accounting for differences in basal immune response. However, this was only a first proof-of-concept cohort study towards the development of a new LTBI detection paradigm as it focused on a limited number of cytokines and a relatively small population of research subjects.

**Figure 1:**
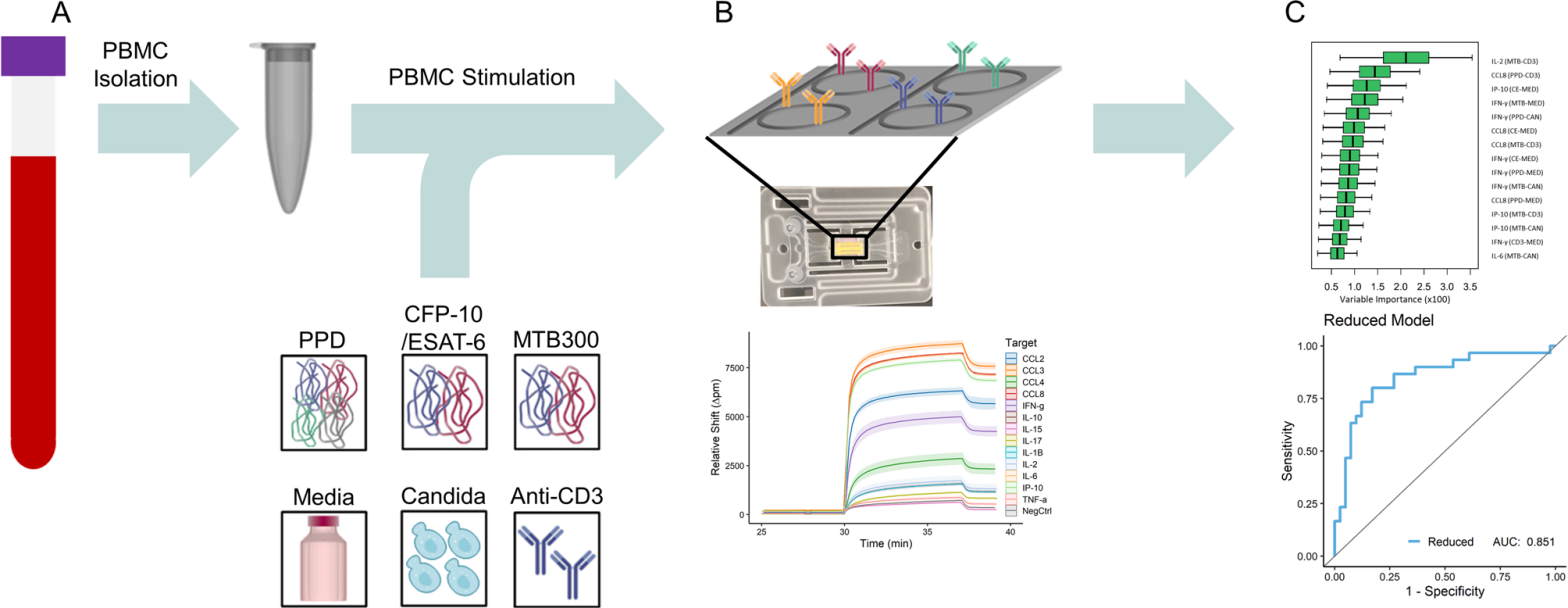
Workflow for LTBI supernatant sample analysis. (**A**) Subject PBMCs are stimulated under multiple on- and off-target conditions. (**B**) Samples are analyzed using the Genalyte Matchbox system, which uses plug-and-play chip and device interfaces to measure cytokine concentrations quickly and reproducibly in a multiplexed assay format. The resonance shift output is recorded and converted to concentrations based on individual cytokine calibrations in the sample matrix. (**C**) Random Forest bioinformatics determine what clinical features are essential for categorical distinctions and predictive accuracy based on variable importance metrics, with statistical data reduction methods employed to identify biomarker signatures most highly correlated with given clinical determinants.

Building upon this previous cohort study, we have now expanded to a larger panel of cytokines (13), again detected using silicon photonic microring sensor arrays, and enrolled a larger research cohort (65 subjects, 75 unique samples). We also have added a new antigen condition with the MTB300 reagent, which is a comprehensive “megapool” of *Mycobacterium tuberculosis* (Mtb) peptides that captures a large fraction of the *Mtb*-specific T cells^16^. Cytokine concentrations were determined for each stimulation condition and normalized by pairwise subtraction of levels from other stimulation conditions. The resulting normalized cytokine levels were then subjected to Random Forest feature selection to identify biomarker signatures correlating with LTBI + status and High Risk of reactivation (the entire workflow is illustrated in Fig. 1). Statistically-driven thresholding was then applied to identify the most relevant biomarkers having the highest predictive accuracy for these clinical designations. Receiver-Operator Characteristic (ROC) curves were generated for both full and reduced data sets with Area-Under-the-Curve (AUC) analyses revealing predictive accuracies exceeding 80% for reduced biomarker signatures. These studies suggest that multiplexed analyses and precision cytokine normalization can generate highly predictive signatures for LTBI status and reactivation potential that may find utility in clinical management of patients with LTBI through a personalized medicine approach.

## Methods

### Reagents and buffers

Reagents, including Dulbecco’s phosphate buffered saline (PBS), bovine serum albumin (BSA), (3-Aminopropyl)triethoxysilane, glycerol, bis(sulfosuccinimidyl)suberate, starting block blocking buffer, Pierce high sensitivity streptavidin-HRP (SA-HRP), 4-chloronaphthol (4-CN), and Drycoat assay stabilizer were purchased from commercial vendors as listed in Table S1. Vendors and catalog numbers for antibodies against all cytokines and the mouse IgG isotype control are summarized in Table S2. Running buffer for all assays was 0.5% BSA in 1X PBS, pH 7.4.

### Cell culture and antigen stimulations

Cell culture and antigen stimulation methods have been described previously^15,17^. Briefly, PBMCs were separated by from whole blood by Ficoll separation and the pellets frozen with 10% DMSO in cell media in liquid nitrogen. PBMC pellets were thawed (viability ≥ 85%) and 1 million cells per mL were stimulated for 40–48 h with either TB-relevant (PPD [10 µg/mL], CFP-10 [4 µg/mL]/ESAT-6 [2 µg/mL], or MTB300 [1 µg/mL]) or off-target (Media—negative control, Candida—off-target control [2 µg/mL], anti-CD3—positive control [200 ng/mL]) antigens^17^. Supernatants from stimulated PBMCs were stored at − 80 °C and shipped on dry ice for cytokine measurements using the 13-plex antigen immunoassay on the microring resonators. Samples were thawed and vortexed prior to loading into a 96-well plate for microring assays. Each sample was analyzed undiluted and after a tenfold dilution in running buffer.

### Clinical category determination

This study was approved by the Mayo Clinic Institutional Review Board and Olmsted County Public Health Services (reference number: 09-003253). All study participants signed an informed written consent and were enrolled in Rochester, MN between July 2017 and December 2018. Sample collection and all experiments were performed in accordance with relevant guidelines and regulations. Study subjects included unexposed individuals and subjects with various risk for TB infection, including untreated LTBI patients and patients with having had LTBI therapy and thus at low risk of reactivation (Table 1). Risk factors for TB infection, TB progression, and/or TB reactivation were extracted through a validated questionnaire and review of medical records as previously described^15,18^. LTBI diagnoses were made per the Center for Disease Control and Prevention (CDC) criteria and based on TB risk factors, and by prior TST and QuantiFERON-TB Gold In-Tube (QFT; Qiagen, Germantown, MD) results^19^. A modified multifactorial predictive modeling platform (i.e. ‘Online TST/IGRA interpreter’), adjusted by LTBI treatment effect, was also applied to estimate the cumulative risk of TB reactivation in all subjects as previously described^17,20^.

**Table 1:** Clinical characteristics for the study cohort.

Group	All	Controls^a^	TST + ^b^	QFT + ^c^	LTBI + ^d^	High Risk +
N (%)	75 (100)	25 (33.3)	37 (49.3)	32 (42.7)	32 (42.7)	24 (32)
Male, N (%)	24 (32)	5 (20.0)	16 (43.2)	13 (40.6)	14 (43.8)	11 (45.8)
Female, N (%)	51 (68)	20 (80.0)	21 (56.8)	19 (59.4)	18 (56.2)	13 (54.2)
HCW, N (%)	55 (73.3)	11 (44.0)	37 (100)	26 (81.3)	27 (84.4)	18 (75)
Age (mean years ± SD)	53.2 ± 17.5	58.5 ± 16.0	49.6 ± 18.4	46.3 ± 17.0	48.6 ± 19.1	45.7 ± 18.6
Predicted risk (mean ± SD)	2.7 ± 7.3	0 ± 0	2.9 ± 3.0	4.8 ± 10.6	3.0 ± 2.5	6.3 ± 11.9

Abbreviations—N (number), HCW (health care worker), SD (standard deviation), TST (Tuberculin Skin Test), QFT (QuantiFERON Gold TB In-Tube test). Cumulative predicted risk of TB reactivation was based on a modified multifactorial modeling platform (i.e. ‘Online TST/IGRA interpreter’) applied to all subjects as previously described^19,20^. All clinical variables are aggregated by positive tests or indications. Study subjects included 5 patients with non-HIV immunosuppressed conditions (one on methotrexate for rheumatoid arthritis, one on sirolumus for lymphangioleiomyomatosis, one with history of chemotherapy and stem-cell transplantation for angioimmunoblastic lymphoma, one on 50 mg daily of prednisone for bullous pemphigoid, and one on hydroxychloroquine and low-dose prednisone for lichenoid mucositis). The total sample set is 75 samples, encompassing 65 unique subjects and 10 additional time points separated by 5–11 months in testing, representing unique samples.

^a^Twenty-five samples from 23 unique unexposed subjects with negative QFT results.

^b^Study cohort includes 4 subjects with unavailable TST results, which were not included in the TST + group estimates.

^c^Study cohort includes 2 subjects with indeterminate QFT results, which were not included in the QFT + group estimates.

^d^LTBI clinical designation was based on current diagnostic guidelines with positive QFT and/or TST results^18^.

### Multiplexed immunoassay instrumentation and assay design

Microring immunoassays were performed on the Maverick Matchbox system (Genalyte, Inc., San Diego, CA) as previously described^21^. This platform utilizes injection-molded microfluidic devices to introduce fully automated flow for all assay steps across functionalized sensor arrays. These devices are disposable, to ensure no contamination between assays^22,23^. Microring arrays were batch functionalized via spotting to yield a 13-plex array of capture antibodies covalently immobilized on sensor substrates. After flowing the sample across the chip, a cocktail containing all the tracer antibodies was flowed across the array followed by signal enhancement reagents. Antibody capture and tracer concentrations used are listed in Table S3. Shifts in resonance wavelength from the signal enhancement step are directly correlated to the concentration of target analytes in solution. Immunoassays were performed with a consistent 30 μl/min flow rate for all steps. There was an initial rinse of 5 min with the running buffer to ensure equilibration of the chip prior to sample analysis. The assay included steps as follows: (1) running buffer (2 min); (2) sample (7 min); (3) running buffer rinse (2 min); (4) biotinylated tracer antibodies (7 min); (5) running buffer rinse (2 min); (6) SA-HRP (7 min); (7) running buffer rinse (2 min); (8) 4-CN (7 min); (9) running buffer rinse (2 min). The total assay time was 38 min. Figure S1 shows a real-time trace of resonance wavelength shifts during a representative multiplexed immunoassay.

### Calibrations and sample analyses

Immunoassays were simultaneously calibrated for all antigens in a multiplexed format. Serial dilutions from a saturating antigen concentration for each multiplexed immunoassay yielded eight-point calibrations relating relative resonance shifts to standard concentrations. To quantify relative shifts, the signal during the buffer rinse before the assay enhancement step (t = 29 min) was subtracted from the final assay rinse step (t = 38 min). Net resonance wavelength shifts ($\Delta pm$) were plotted as a function of standard concentration and fit to a four-parameter logistic function as described previously^21^ via the following equation:

1 $$ \Delta pm = A_{2} + \frac{{\left( {A_{1} - A_{2} } \right)}}{{1 + \left( {\frac{{\left[ {antigen} \right]}}{{\left[ {antigen} \right]_{0.5} }}} \right)^{p} }} $$

where $A_{1}$ is the lower resonance shift bound, $A_{2}$ is the upper resonance shift bound, $\left[ {antigen} \right]_{0.5}$ is the concentration yielding 50% of maximum signal, and $p$ is the power parameter affecting the slope at $\left[ {antigen} \right]_{0.5}$. Limits of detection (LOD) and quantification (LOQ) were defined as the blank signal plus 3 times and 10 times the standard deviation of the blank, respectively. Each calibration was performed in triplicate for each sample dilution (Fig. S2) as measured with 4 sensors per technical replicate.

Samples were analyzed undiluted (1X) and diluted (0.1X) into running buffer. Cytokine concentrations within supernatant samples were determined from corresponding serum calibrations (10% and 1% serum, respectively, matching the serum content in supernatant samples). Final concentrations were measured using the most appropriate dilution/calibration as determined by how close the value came to the midpoint of the calibration. Precision normalization was achieved by separately subtracting the control or off-target cytokine concentration from that measured at the other stimulation conditions.

### Random forest and ROC curve analyses

Random Forest methods were utilized to determine the biomarker features associated with both LTBI diagnosis (“LTBI + ”) and increased risk of reactivation (“High Risk”) clinical designations^24^. Random Forest is an ensemble classification algorithm that can detect nonlinear effects of covariate features. Moreover, it provides a ranking of importance of each covariate, while the importance is identified by their effect on classification of either LTBI or risk of reactivation. Using these randomized decision tree outputs, a ROC curve can be established that indicates the predictive accuracy the variables hold for a given classification.

Feature importance analysis was done for all variables available (143 total), and ROC curves were produced. Utilizing the important features from those analyses, a secondary Random Forest algorithm was produced for further enhancement of predictive accuracy by removing unimportant or noisy features. This was done identically for both LTBI + and High Risk clinical designations. Mann–Whitney plots were utilized to evaluate each important variable in the signature. All informatics and plotting were performed using R coding language^25,26^.

## Results

### Study subjects

We sequentially enrolled a total of 65 subjects, including 32 subjects with diagnosis of LTBI by QFT and/or TST results and 5 immunosuppressed patients for various medical conditions. The total sample set included 75 samples, encompassing 65 unique subjects and 10 additional time points separated by 5–11 months in testing, representing unique samples (Table 1). All HIV-tested subjects were non-reactive by ELISA (45 out of 65). The majority of study participants were health care workers (73%) with various risks of TB exposure and 10 (15.4%) had prior history of BCG vaccination. Twenty-three subjects with negative QFT results and a predicted cumulative TB risk of zero were included as unexposed control subjects (Table 1). Eight individuals who previously received treatment for LTBI and were considered at low risk for TB reactivation. There were no significant age differences across the study clinical designations.

### Full versus reduced random forest feature selection

**Figure 2:**
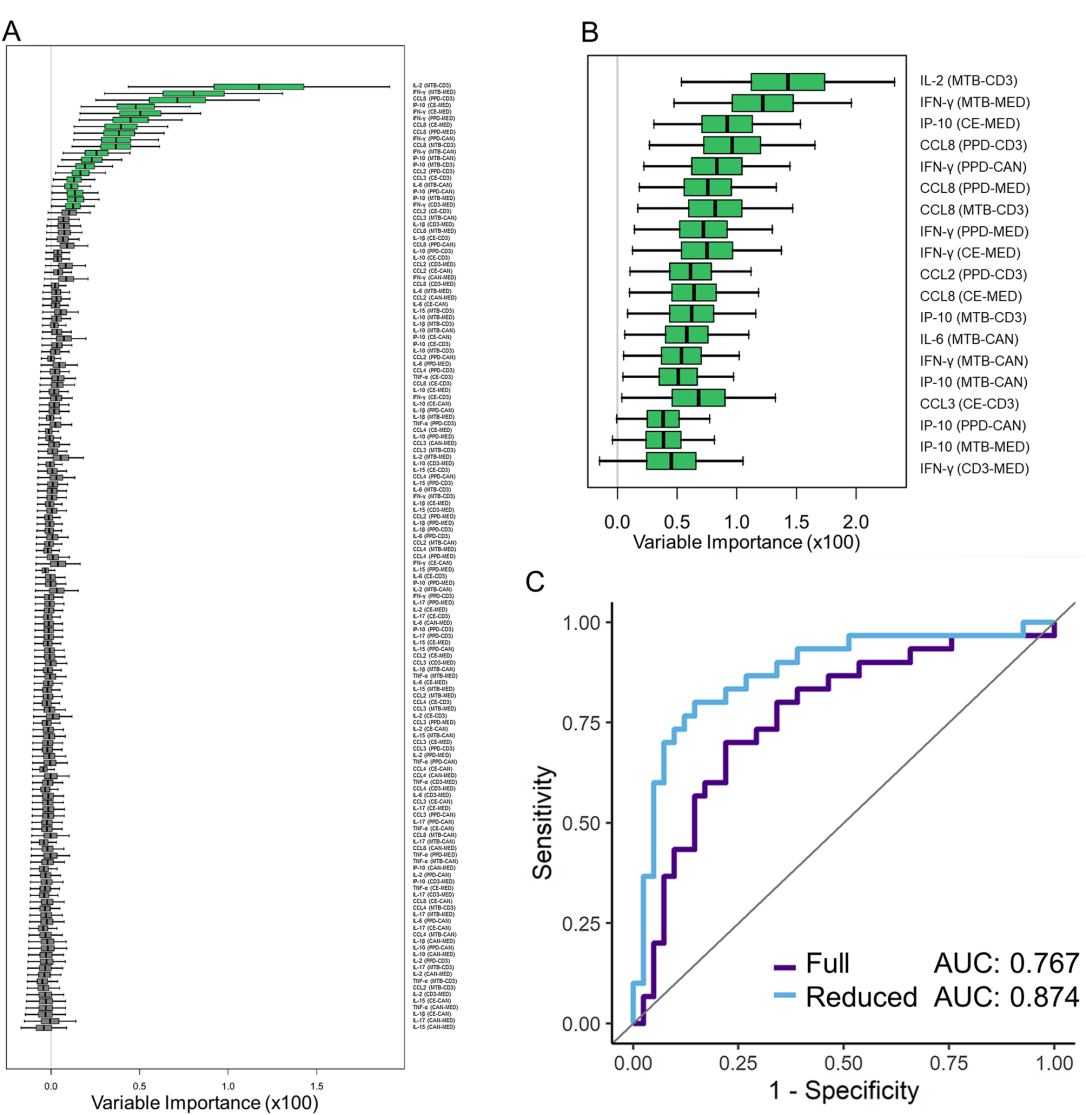
Comparison of (**A**) full random forest feature and (**B**) the threshold-based reduced random forest feature analysis for the LTBI + clinical category. Features for the reduced analysis are determined by Variable Importance (VIMP) metrics. (**C**) ROC Curves for full and reduced analysis represent the predictive power of each method, with AUC values corresponding to the percent predictive accuracy. Notably, the reduced biomarker set offers improved predictive accuracy. Abbreviations—MED (cell media), CAN (Candida), CD3 (anti-CD3), PPD (purified protein derivative), CE (CFP-10/ESAT-6), MTB (MTB300). Normalized conditions are denoted as Condition 1 minus Condition 2 (i.e. PPD-MED is the PPD condition minus the negative control cell media condition).

The initial Random Forest analysis was performed using all possible normalized features from the full dataset (13 cytokines × 11 normalized, pairwise conditions), identifying 143 features viable for the LTBI + clinical designation. Based on all possible features, a ROC curve for LTBI + was produced (Fig. 2A), yielding an AUC of 0.767 (76.7% predictive value). From this full feature analysis, a reduced random forest validation containing all the important features from the full analysis was created using the statistical variance of each feature and establishing a threshold based on predictive accuracy improvement. Therefore, the 19 identified features after data reduction, which span a variety of cytokines and normalized conditions, can be considered as the most relevant to the ROC curve’s predictive power (Fig. 2B). A ROC curve constructed using only these reduced features yielded an AUC of 0.874 (Fig. 2C). Mann–Whitney analyses of the statistical significance of variables included in the reduced analysis are detailed in Table S4.

### Importance of MTB300 stimulation condition

**Figure 3:**
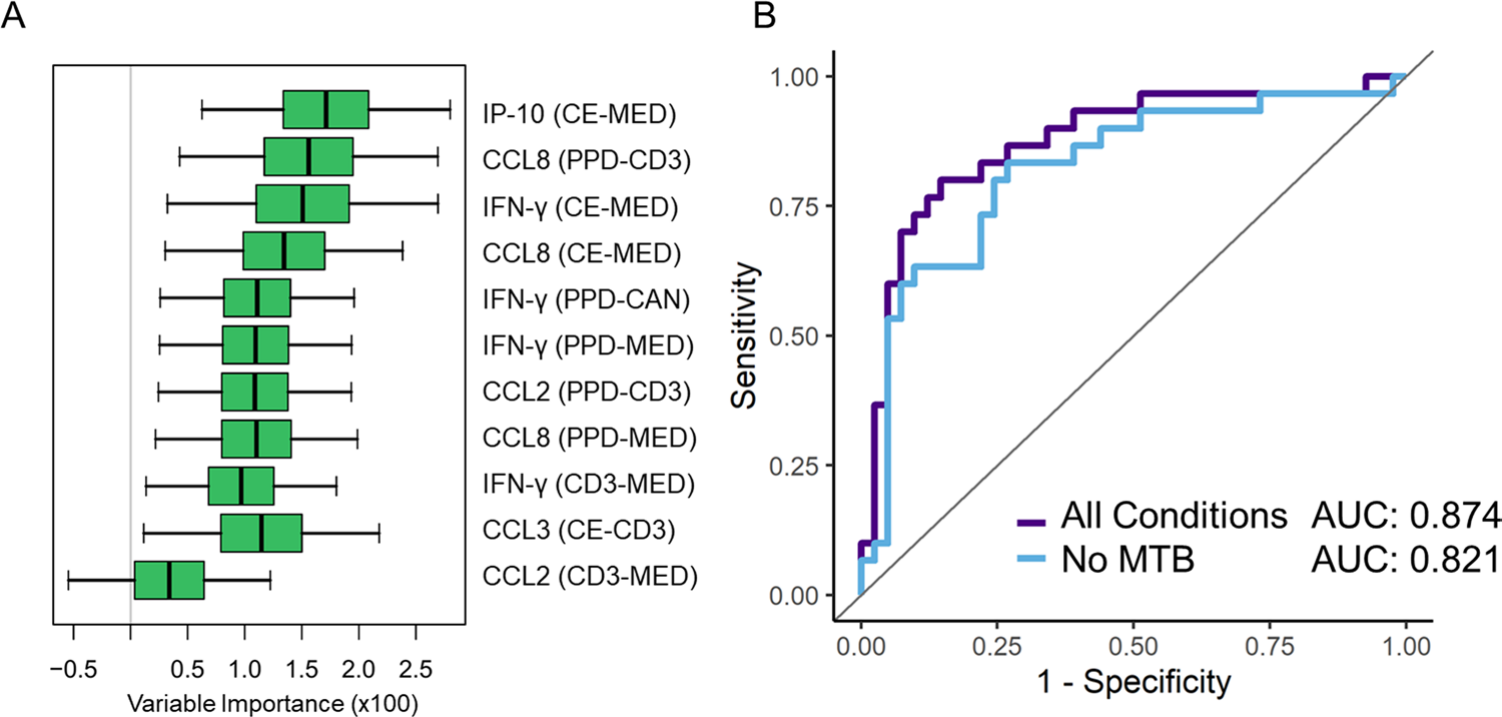
(**A**) Reduced random forest feature analysis for the LTBI + clinical designation when the MTB stimulation is removed. (**B**) ROC curve comparison of LTBI + reduced random forest with and without MTB stimulation condition. This indicates an improved predictive accuracy through the inclusion of the MTB stimulation condition.

To assess the added benefit of the MTB300 stimulation condition, which specifically targets a large fraction of *Mtb*-specific CD4^+^ and CD8^+^ T cell populations, separate Random Forest feature selection and data reduction algorithms were performed for LTBI + in the absence of any normalized conditions that included the MTB300 stimulation (Fig. 3A). A quantitative comparison between the previous reduced feature set for LTBI and the further reduced analysis without MTB300 shows a loss of 5.3% in prediction accuracy (AUC changed from 0.874 to 0.821), indicating a valuable role for MTB300 in improving LTBI diagnostic utility (Fig. 3B).

### Stratification of subjects based upon high risk of reactivation

**Figure 4:**
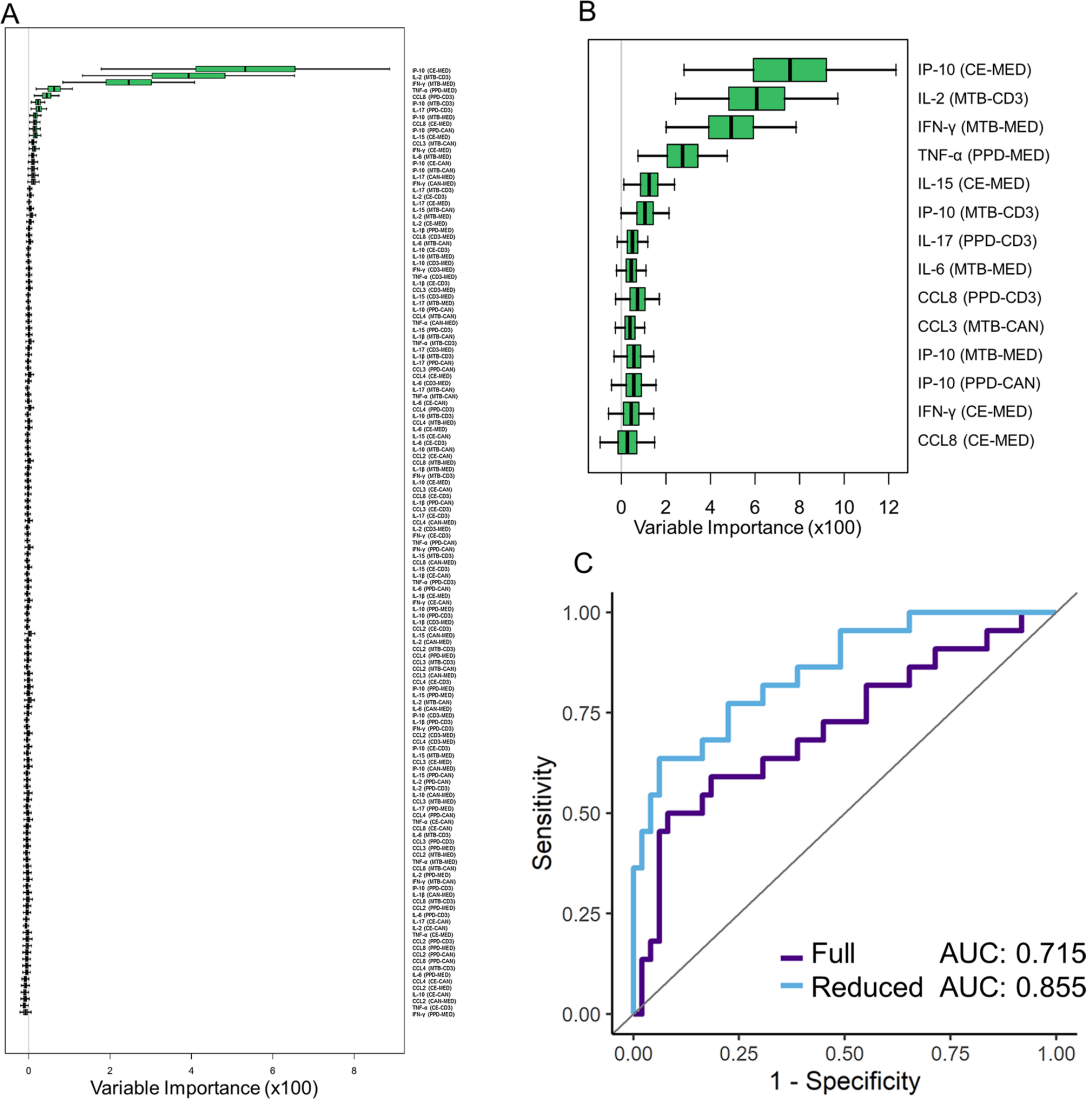
Comparison of (**A**) full random forest feature and (**B**) the threshold-based reduced random forest feature analysis for the High Risk clinical designation. (**C**) ROC Curves for full and reduced analysis show an increase in predictive accuracy for the reduced feature set.

Reduced Random Forest analysis was performed using the entire normalized dataset as input for the High Risk of reactivation clinical designation. Though this subpopulation is smaller than the LTBI + pool, it is still large enough for robust feature identification. The full data set analysis for High Risk used all 143 features with a resulting AUC of 0.715 (Fig. 4A). Upon performing the statistical feature reduction, 14 features were identified as most relevant, and these reduced features yielded a predictive accuracy of 0.855 (Fig. 4B, C). Mann–Whitney analyses of the statistical significance of these reduced biomarkers is detailed in Table S5.

## Discussion

LTBI is a persistent and quiescent infection that has proven difficult to identify accurately using conventional diagnostic methods. The added stratification of progression and/or reactivation potential has further been a diagnostic challenge that limits the ability to robustly identify patients most likely to benefit the most from antibiotic treatment. Utilizing our 13-target multiplexed cytokine panel and powerful bioinformatic approaches, we have identified multi-biomarker signatures that show strong correlations to clinical designations of LTBI + and High Risk of reactivation with an approach that only uses input biomarker levels to achieve these diagnoses.

Based upon our previous study, we found that precision normalization revealed a richer biomarker signature compared to feature selection using non-normalized cytokine levels within stimulated PBMC supernatants. We attributed this to elimination of patient-to-patient heterogeneities in basal immune responses, which were corrected by subtracting non-TB control levels. Therefore, we utilized normalized cytokine levels obtained by subtracting control and off-target antigen stimulation conditions from other supernatant solutions. Using these normalized values as input, iterative feature selection methods were applied as described to identify biomarkers that first correlated with the LTBI + designation, as determined via extensive clinical chart review. Using all 143 possible features from the full dataset (Fig. 2A), a ROC curve was generated and found to have an AUC of 0.767, or a 76.7% predictive accuracy (Fig. 2C). Realizing that some features might have more overall significance than others, a statistical data reduction routine was performed, identifying 19 features that most strongly correlated with LTBI + (Fig. 2B). Biomarkers in the reduced set included IL-2, IFN-γ, IP-10, CCL8, CCL2, IL-6, and CCL3 under varying normalized stimulation conditions. The ROC/AUC analysis using just the reduced feature showed a predictive accuracy of 87.4%, which was a 10.7% improvement over the full feature set (Fig. 2C). We hypothesize that some of the features in the full data set feature reduction, while weakly correlated with LTBI + , contained higher levels of biological variance that reduce the overall predictive potential across the diverse LTBI + cohort. Application of this thresholding approach to data reduction focused the biomarker signature on those features most relevant, thereby increasing the predictive accuracy of the ROC curve and diagnostic efficacy. For future studies, this realization is also important as it will allow for the development of more focused and simplified cytokine panels that do not require non-essential biomarkers.

Beyond the inclusion of more cytokines in this updated panel, we also included the MTB300 stimulation condition, so as to probe the relative value of this stimulation condition in more accurately diagnosing LTBI. MTB300 is a mixture of 300 Mtb-derived T cell epitopes that specifically targets a large fraction of *Mtb*-specific CD4^+^ and CD8^+^ T cells^16,27^. Both Purified Protein Derivative (PPD) and CFP-10/ESAT-6 (CE) have traditionally been used in TST and QFT testing, respectively. While these stimulations can elucidate an immune response that is indicative of tuberculosis exposure, they are potentially insufficient to differentiate LTBI or reactivation risk. In addition, the majority of the human response to peptide antigens from *Mtb* is not contained in the peptide mixtures of IGRAs^16^. In this context, the use of the MTB300 “megapool” of peptides in our immune profiling method may theoretically confer a better sensitivity over IGRA methods by targeting a larger fraction of the *Mtb*-specific T cells. Importantly, MTB300 contains 300 primarily MHC class II restricted T cell epitopes from 90 different antigens in Mtb. While these epitopes are derived from Mtb many of these epitopes are also conserved in the *M. bovis* bacillus Calmette-Guérin (BCG) and also non-tuberculous mycobacteria. To verify that the new stimulation condition adds value to our multiplexed cytokine profiling approach, we performed identical feature selection and data reduction methods omitting all normalized features that contained the MTB condition. Focusing just on the reduced feature sets for LTBI + for comparison (Fig. 3), we found that AUCs fell by 5.3% upon the removal of the MTB condition, indicating the importance of MTB300 for improved diagnostic accuracy.

Similarly to the LTBI + designation, we also performed identical feature selection of both the full normalized feature set and after data reduction for the High Risk clinical designation (Fig. 4). Again, the reduced data set yields an improved predictive value (85.5% reduced vs. 71.5% full). Additionally, we find a similar number of relevant biomarker features for High Risk and LTBI + designations, with IFN-γ, IP-10, IL-2, IL-6, CCL3, and CCL8 appearing in both designations. These targets are important in T cell recruitment, granuloma formation, and inflammatory regulation^28–32^, and so the overlapping relevance for these biomarkers is not unsurprising^9,10,33^. Interestingly, we also find some markers that are unique between the designations. Specifically, the chemotactic marker CCL2, which is induced by tissue injury or infection^34^, was identified as relevant for LTBI + , but not for High Risk. Conversely, the cytokines TNF-α, IL-17, and IL-15 were found to be uniquely relevant for the High Risk designation^35–38^. Given that LTBI reactivation is thought to occur due to global changes to host immune regulation, it is intriguing that cytokines not typically associated with the acute TB infection response to have diagnostic utility in reactivation risk stratification. As a corollary, patients on anti-TNF-α therapies are known to have higher risk of reactivation, which suggests the potential for diagnostic monitoring of multiple immune regulatory factors when surveilling for reactivation risk.

Overall, we found that this multiplexed, precision normalization approach to diagnosing LTBI and stratifying high reactivation potential resulted in multi-biomarker signatures with AUCs in excess of 0.80 for reduced feature sets. This study highlights the critical value in moving beyond single biomarker-based methods, such as IGRAs that just analyze for IFN-γ. Furthermore, we found that MTB300 provides a measurable improvement in the predictive accuracy of the LTBI + diagnostic signature. It also validates our platform as a potential point-of-care system with fast response times for clinical implementation.

Our study does have some potential limitations. Specifically, there is no diagnostic gold standard for LTBI, and available diagnostic tests are imperfect. However, our study subjects were carefully selected to minimize heterogeneity in the study groups and to align with current clinical diagnostic standards^18^. In addition, most of our study cohort included non-HIV immunocompetent individuals, which could limit the applicability of our study findings to immunosuppressed subjects and people with HIV infection. Another potential limitation is the use of the ‘Online TST/IGRA interpreter,’ which has not been prospectively validated. However, we decided to use this predictive tool for research purposes in absence of any other unbiased clinical tool that theoretically quantifies cumulative risk of TB reactivation in LTBI using TST and/or IGRA results plus evaluation of an individual’s most relevant epidemiologic and clinical characteristics^20^. Moreover, the annual risk of infection estimated by the ‘Online TST/IGRA interpreter’ is based on long-term longitudinal data of TB reactivation^39^. We also understand that without a direct comparison to a cohort of active TB patients, we are missing potential classifiers to understand progression to an active TB disease. While we enrolled some patients with active TB, there were too few to statistically compare with our High Risk designation signature, and we plan to further study this important cohort in the future. Lastly, nine study subjects participated twice several months after their initial study participation; however, we decided to include their study data since some of them had changes their follow-up QFT results, and thus their LTBI diagnostic designation. These immune profiling changes probably represent the dynamic nature of LTBI in some of these subjects, which we wanted to also capture during this study.

## Conclusion

Using this expanded 13-plex cytokine immunoassay biosensor panel, we have demonstrated the ability to identify complex biomarker signatures for LTBI status and to elucidate targets of interest for reactivation risk assessment. Our bioinformatics approach using Random Forest feature selection has shown excellent utility in determining important biomarkers and normalized conditions vital for clinical discrimination between subjects. Furthermore, the feature reduction leads to an improved predictive accuracy and the potential to optimize the multiplexed panel to its most essential components for simplified assay development. We found multiple markers that are essential for both LTBI and reactivation risk, including IFN-γ, IP-10, IL-2, IL-6, CCL3, and CCL8, which represents a broader immunological profile than single target assays. Conversely, distinctive targets such as CCL2 reveal subjects at higher risk of reactivation from the greater LTBI + cohort. We also revealed the value of the peptide pool MTB300 for future stimulated cell-based analyses through data reduction methods. Predictive accuracies for both designations were above 80%, which indicates the potential for improved patient monitoring and clinical care. This biosensor technique, combined with robust bioinformatics, supports this unique strategy for biomarker-only signatures yielding reliable evidence-based clinical decisions for LTBI and the reactivation risk.

## Supplementary Information

Supplementary Information 1.
