# Supplementary material for: Risk assessment of latent tuberculosis infection through a multiplexed cytokine biosensor assay and machine learning feature selection
Source: Sci Rep. 2021 Oct 15;11:20544. doi: 10.1038/s41598-021-99754-3 (PMC8520014; doi:10.1038/s41598-021-99754-3)
Supplement: Supplementary file 1 — Supplementary Information 1. [file 41598_2021_99754_MOESM1_ESM.docx]

Supporting information for

**Risk Assessment of Latent Tuberculosis Infection through a Multiplexed Cytokine Biosensor Assay and Machine Learning Feature Selection**

Heather M. Robison,^1,*^ Cole A. Chapman,^1,*^ Haowen Zhou,^2^ Courtney L. Erskine,^3^ Elitza Theel,^4^ Tobias Peikert,^5^ Cecilia S. Lindestam Arlehamn,^6^ Alessandro Sette,^6,7^ Colleen Bushell,^8^ Michael Welge,^8^ Ruoqing Zhu,^2^ Ryan C. Bailey,^1,†^ Patricio Escalante^5,†^

^1^Department of Chemistry, University of Michigan, 930 North University Avenue, Ann Arbor, MI

^2^Department of Statistics, University of Illinois Urbana-Champaign, 725 South Wright Street, Champaign, IL

^3^Department of Immunology, Mayo Clinic, 200 First Street SW, Rochester, MN

^4^Department of Laboratory Medicine, Mayo Clinic, 200 First Street SW, Rochester, MN

^5^Division of Pulmonary and Critical Care Medicine, Department of Medicine, Mayo Clinic, 200 First Street SW, Rochester, MN

^6^Division of Vaccine Discovery, La Jolla Institute for Immunology, La Jolla, CA

^7^Department of Medicine, University of California San Diego, La Jolla, CA

^8^National Center for Supercomputing Applications, University of Illinois at Urbana-Champaign, 1205 W. Clark St., Urbana, IL

*,^†^ Equal contributions

**TABLE OF CONTENTS**

**Table S1.** Buffers and General Reagents...…………………………………………........S-2

**Table S2.** Antibodies and Recombinant Antigens..……………………………………....S-3

**Table S3.** Immunoassay Antibody Concentrations………..…………………….……….S-4

**Figure S1.** Representative Multiplexed Immunoassay Microring Resonator Trace.….S-5

**Figure S2.** Simultaneous Complex Matrix Panel Calibrations…………….………........S-6

**Table S4.** Mann-Whitney Results for LTBI+ Clinical Designation……………..………..S-7

**Table S5.** Mann-Whitney Results for High Risk Clinical Designation ……………........S-8

**Table S1.** Reagents for buffers, chip functionalization and storage, and immunoassay steps.

| **Reagent** | **Source** | **Catalog Number** |
| --- | --- | --- |
| Dulbecco’s phosphate buffered saline | Millipore Sigma | D5573 |
| Bovine serum albumin | Millipore Sigma | A2153 |
| (3-Aminopropyl)triethoxysilane | Millipore Sigma | 440140 |
| Glycerol | Thermo Fisher Scientific | BP229 |
| bis(sulfosuccinimidyl)suberate | Thermo Fisher Scientific | A39266 |
| starting block blocking buffer | Thermo Fisher Scientific | 37538 |
| Pierce high sensitivity streptavidin-HRP | Thermo Fisher Scientific | 21130 |
| 4-chloronaphthol | Thermo Fisher Scientific | 34012 |
| Drycoat assay stabilizer | Virusys Corporation | AG066 |

**Table S2.** Antibodies and recombinant standard proteins used in the multiplexed immunoassay.

| **Target** | **Role** | **Source** | **Catalog Number** |
| --- | --- | --- | --- |
| CCL2 | Capture  Antigen  Tracer | Thermo Fisher  Thermo Fisher  Thermo Fisher | 14-7099  14-8398  13-7096 |
| CCL3 | Capture  Antigen  Tracer | R&D Systems  R&D Systems  R&D Systems | MAB670-100  270-LD-010  MAB270-100 |
| CCL4 | Capture  Antigen  Tracer | R&D Systems  R&D Systems  R&D Systems | CUSTOI702-AZY021708A  271-BME-010  CUSTOI702-IGH021710A |
| CCL8 | Capture  Antigen  Tracer | R&D Systems  R&D Systems  R&D Systems | MAB281-100  281-CP-010  BAF281 |
| IFN-g | Capture  Antigen  Tracer | Mabtech  Thermo Fisher  Mabtech | 3420-3-250  BMS303  3420-6-250 |
| IL-1b | Capture  Antigen  Tracer | Thermo Fisher  Thermo Fisher  Thermo Fisher | 14-7018-85  RIL1BI  13-7016-85 |
| IL-2 | Capture  Antigen  Tracer | BD Biosciences  Thermo Fisher  BD Biosciences | 555051  14-8029  555040 |
| IL-6 | Capture  Antigen  Tracer | Thermo Fisher  Thermo Fisher  Thermo Fisher | 16-7069  14-8069  13-7068 |
| IL-10 | Capture  Antigen  Tracer | Thermo Fisher  Thermo Fisher  Thermo Fisher | 16-7108  14-8109-80  13-7109 |
| IL-15 | Capture  Antigen  Tracer | R&D Systems  R&D Systems  R&D Systems | MAB647  247-ILB-005  BAM247 |
| IL-17 | Capture  Antigen  Tracer | Mabtech  Mabtech  Mabtech | 3520-3-250  3520-10  3520-6-250 |
| IP-10 | Capture  Antigen  Tracer | BD Biosciences  BD Biosciences  BD Biosciences | 555046  551130  555048 |
| TNF-a | Capture  Antigen  Tracer | Biolegend  Biolegend  Biolegend | 502802  570102  502904 |

**Table S3.** Antibody concentrations used for capture and tracer pairs. Captures were spotted in 1xPBS, 5% glycerol. All tracers were diluted in running buffer. Streptavidin-Horseradish Peroxidase (SA-HRP) was diluted to 4μg/mL for all experiments.

| **Target** | **Capture (mg/mL)** | **Tracer (μg/mL)** |
| --- | --- | --- |
| CCL2 | 0.25 | 2 |
| CCL3 | 0.25 | 1 |
| CCL4 | 0.25 | 2 |
| CCL8 | 0.25 | 2 |
| IFN-g | 0.25 | 2 |
| IL-1b | 0.25 | 2 |
| IL-2 | 0.25 | 2 |
| IL-6 | 0.25 | 2 |
| IL-10 | 0.25 | 2 |
| IL-15 | 0.25 | 2 |
| IL-17 | 0.25 | 2 |
| IP-10 | 0.25 | 2 |
| TNF-a | 0.25 | 2 |


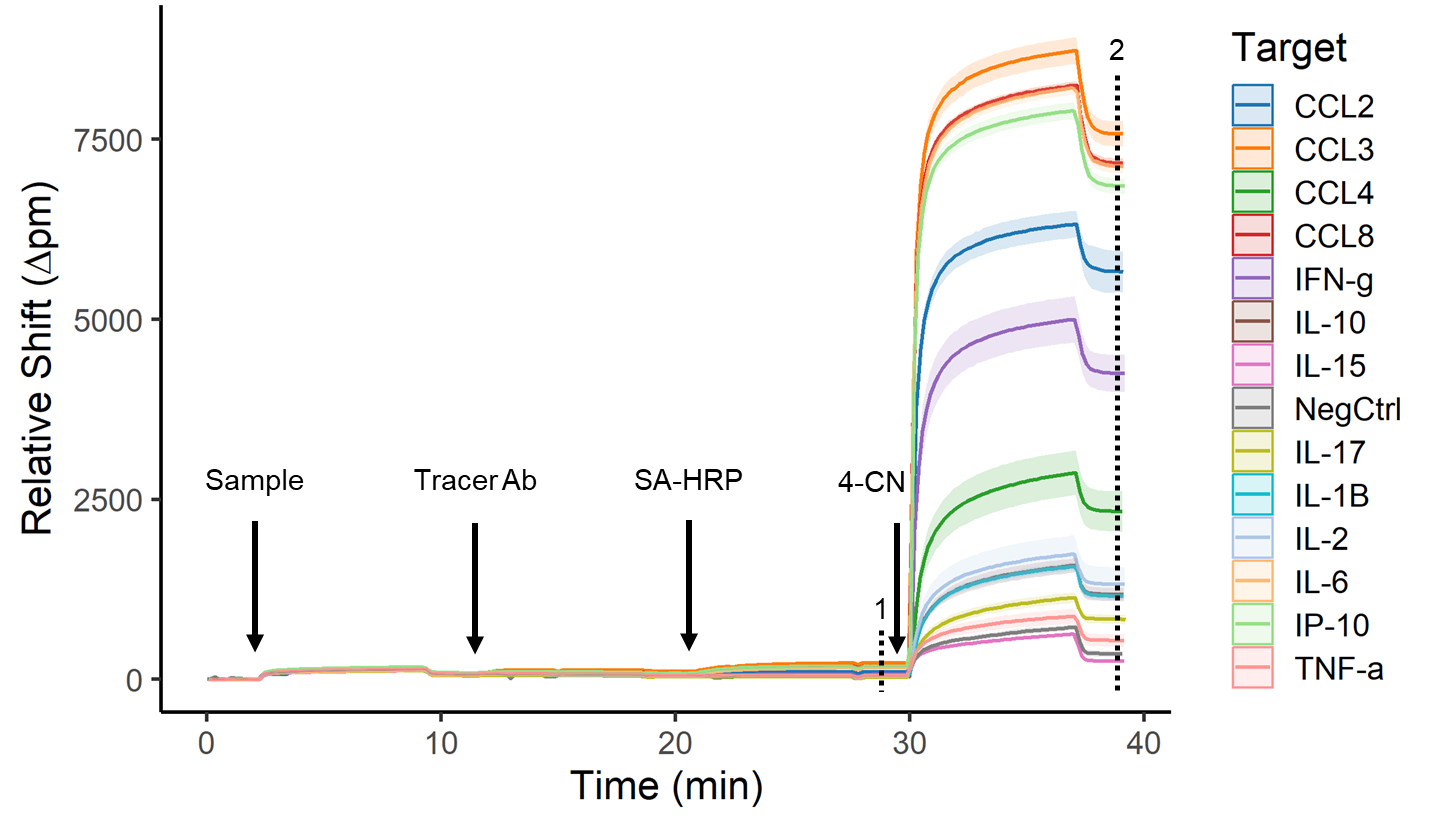


**Figure S1.** Real-time resonance wavelength shifts for a representative multiplexed immunoassay. Two-minute buffer rinses occur between each reagent step. Shaded areas represent the standard deviation across four sensors per target in a single assay. Net shifts are calculated as the difference in signal between the end of the assay (2) and the running buffer rinse signal before amplification (1).


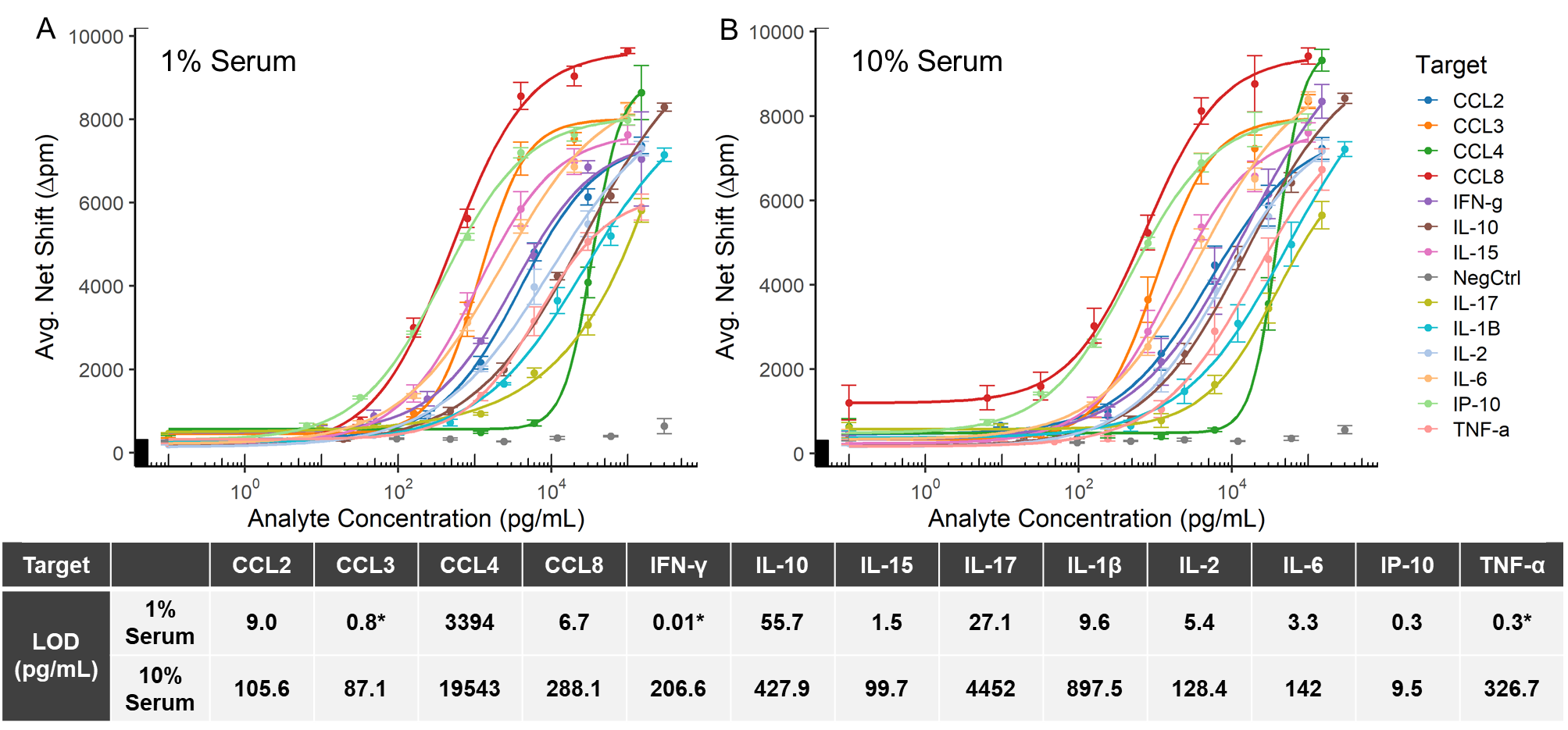


**Figure S2.** Simultaneous multiplexed calibrations on the Genalyte Matchbox platform for A) 1% serum samples and B) 10% serum samples. Error bars represent standard deviation and are from n=3 calibrations, n=4 rings per target. LODs were calculated for each target in each matrix dilution as the blank signal plus three times the standard deviation of the blank. *Values were calculated from the asymptotic minimum of the fit, due to the LOD calculation falling below the fit parameters.

**Table S4.** Mann-Whitney tests for significant features from the reduced random forest analysis of the LTBI clinical category.

| **Condition** | **Target** | **P Value** |
| --- | --- | --- |
| PPD-CD3 | CCL2 | 0.007 |
| CE-CD3 | CCL3 | 0.2 |
| PPD-CD3 | CCL8 | 0.001 |
| PPD-MED | CCL8 | 0.005 |
| MTB-CD3 | CCL8 | 0.001 |
| CE-MED | CCL8 | 0.005 |
| MTB-MED | IFN-γ | 0.0002 |
| PPD-CAN | IFN-γ | 0.5 |
| PPD-MED | IFN-γ | 0.0001 |
| CE-MED | IFN-γ | 0.01 |
| MTB-CAN | IFN-γ | 0.03 |
| CD3-MED | IFN-γ | 0.08 |
| MTB-CD3 | IL-2 | 0.0004 |
| MTB-CAN | IL-6 | 0.07 |
| CE-MED | IP-10 | 0.0004 |
| MTB-CD3 | IP-10 | 0.002 |
| MTB-CAN | IP-10 | 0.003 |
| PPD-CAN | IP-10 | 0.8 |
| MTB-MED | IP-10 | 0.01 |

**Table S5.** Mann-Whitney tests for significant features from the reduced random forest analysis of the High Risk clinical category.

| **Condition** | **Target** | **P Value** |
| --- | --- | --- |
| MTB-CAN | CCL3 | 0.03 |
| PPD-CD3 | CCL8 | 0.02 |
| CE-MED | CCL8 | 0.1 |
| MTB-MED | IFN-γ | 0.0002 |
| CE-MED | IFN-γ | 0.04 |
| CE-MED | IL-15 | 0.2 |
| PPD-CD3 | IL-17 | 0.1 |
| MTB-CD3 | IL-2 | 0.0003 |
| MTB-MED | IL-6 | 0.2 |
| CE-MED | IP-10 | 0.0006 |
| MTB-CD3 | IP-10 | 0.003 |
| MTB-MED | IP-10 | 0.01 |
| PPD-CAN | IP-10 | 0.4 |
| PPD-MED | TNF-α | 0.6 |
